# Prior Exposure to Immunosuppressors Sensitizes Retinal Microglia and Accelerates Optic Nerve Regeneration in Zebrafish

**DOI:** 10.1155/2019/6135795

**Published:** 2019-02-10

**Authors:** Ilse Bollaerts, Jessie Van houcke, An Beckers, Kim Lemmens, Sophie Vanhunsel, Lies De Groef, Lieve Moons

**Affiliations:** Neural Circuit Development and Regeneration Research Group, Department of Biology, KU Leuven, 3000 Leuven, Belgium

## Abstract

As adult mammals lack the capacity to replace or repair damaged neurons, degeneration and trauma (and subsequent dysfunction) of the central nervous system (CNS) seriously constrains the patient's life quality. Recent work has shown that appropriate modulation of acute neuroinflammation upon CNS injury can trigger a regenerative response; yet, the underlying cellular and molecular mechanisms remain largely elusive. In contrast to mammals, zebrafish retain high regenerative capacities into adulthood and thus form a powerful model to study the contribution of neuroinflammation to successful regeneration. Here, we used pharmacological immunosuppression methods to study the role of microglia/macrophages during optic nerve regeneration in adult zebrafish. We first demonstrated that systemic immunosuppression with dexamethasone (dex) impedes regeneration after optic nerve injury. Secondly, and strikingly, local intravitreal application of dex or clodronate liposomes prior to injury was found to sensitize retinal microglia. Consequently, we observed an exaggerated inflammatory response to subsequent optic nerve damage, along with enhanced tectal reinnervation. In conclusion, we found a strong positive correlation between the acute inflammatory response in the retina and the regenerative capacity of the optic nerve in adult zebrafish subjected to nerve injury.

## 1. Introduction

One of the first events manifesting upon central nervous system (CNS) injury is an acute immune response, which comprises the reactivation of resident microglia, and in severe cases also the recruitment of leukocytes from the bloodstream. Leukocyte infiltration is characterized by rapid invasion of neutrophils and monocyte-derived macrophages, while lymphocytes may follow at later stages [1–3]. All of these (re)activated immune cells secrete cytokines and chemokines, thereby modulating their environment and presumably affecting the neurodegenerative but also the regenerative outcome [4, 5]. Indeed, although this acute inflammation has long been considered detrimental for functional recovery, there is now compelling evidence that neuroinflammation may also have beneficial effects – if properly orchestrated.

As an integral part of the CNS, the primary visual pathway has proven to be a powerful model system to study the inflammatory mediator cells affecting CNS repair. In rodent models of optic nerve injury, the induction of a restricted ocular inflammation (e.g., via lens injury or toll-like receptor 2 (TLR2) agonists such as the yeast cell wall extract zymosan or the lipopeptide Pam_3_Cys) has been repeatedly shown to improve survival of retinal ganglion cells (RGCs) after axonal damage and enable them to extend regenerating axons into the optic nerve [6–16]. Although this clearly illustrates that acute inflammation can be proregenerative, the relative contributions of the different inflammatory cell types are not yet fully elucidated, and the exact molecular cues and downstream signalling pathways that mediate the effects of inflammatory stimulation remain to be uncovered.

Unlike mammals, adult zebrafish display a tremendous regenerative capacity, also in the CNS. Upon damage to the optic nerve, most zebrafish RGCs survive and regrow their axons to reconnect with their target areas in the brain, eventually restoring vision [17–21]. Importantly, also in zebrafish, neuroinflammation has been put forward as an important player in successful CNS regeneration [22–24]. Moreover, a high degree of conservation of the (inflammatory) mechanisms regulating optic nerve regeneration has been demonstrated [17, 19, 25–28]. Thus, research in zebrafish can help to improve our understanding of how acute neuroinflammation can be coupled to successful CNS regeneration.

In this study, we aim to shed light on the role of microglia/macrophages during optic nerve regeneration in zebrafish, using immunosuppressive treatments. We used the synthetic glucocorticoid dexamethasone (dex) and clodronate liposomes to pharmacologically deplete microglia/macrophages and investigated how systemic and local application of these drugs affects the regenerative outcome.

## 2. Methodology

### 2.1. Zebrafish Maintenance

Zebrafish (*Danio rerio*) were raised and maintained under standard laboratory conditions, at 27.5°C and on a 14/10-hour light/dark cycle. All experimental procedures were performed in *Tg(coro1a:eGFP; lyz:DsRed)* fish of 5-6 months old. In these fish, enhanced green fluorescent protein (eGFP) is expressed in microglia, macrophages, and neutrophils, while *Discosoma* sp. red fluorescent protein (DsRed) is present in neutrophils only [24, 29]. Of note, the *coro1a* promoter might be active in other leukocytes as well [29], most likely in T lymphocytes. This implies that the eGFP^+^ DsRed^−^ cell population may not consist uniquely of microglia/macrophages, but may include a small percentage of other leukocytes. For all experiments, mixed groups of male and female fish were used. All animal experiments were approved by the KU Leuven Animal Ethics Committee and executed in strict accordance with the European Communities Council Directive of 20 October 2010 (2010/63/EU).

### 2.2. Optic Nerve Crush (ONC)

Optic nerve crush (ONC) was performed as previously described [20, 21, 30]. Briefly, zebrafish were anesthetized in 0.02% buffered tricaine (MS-222, Sigma-Aldrich) and placed under a dissection microscope (Leica, Deerfield, IL). Using sterile forceps (Dumont No. 5, FST), the connective tissue around the left eye was removed. The eye was gently lifted out of its orbit to expose the optic nerve. Then, the nerve was crushed for ten seconds, at a distance of 500 *μ*m from the optic nerve head. Care was taken not to damage the ophthalmic artery running parallel to the nerve. After surgery, fish were returned to separate tanks for recovery.

### 2.3. Immunosuppressive Treatments

#### 2.3.1. Systemic Immunosuppression

To obtain systemic immunosuppression, the synthetic glucocorticoid dex was used as previously described [22]. Briefly, dex (Sigma-Aldrich, Cat. No. D1765) was dissolved in methanol and added to the tank water, at a concentration of 15 mg/L. Control fish received an equal volume of methanol. Tank water was refreshed daily. The treatment was started 2 weeks before ONC and was continued until the end of the experiment, to ensure efficient depletion throughout the entire experiment.

#### 2.3.2. Local Immunosuppression via Intravitreal Injections

For local immunosuppression, intravitreal injections were performed as previously described [20]. Briefly, fish were anesthetized in 0.02% buffered tricaine (MS-222, Sigma-Aldrich) and placed under a dissection microscope with the left eye facing upward. Using a microinjector (UMP3, World Precision Instruments, New Haven, CT), fish were intravitreally injected with 300 nL of dex (1.65 mg/mL in sterile 0.68% saline; Sigma-Aldrich, Cat. No. D2915), clodronate liposomes (http://ClodronateLiposomes.com, used undiluted as supplied), or 0.68% saline (the vehicle for both). Depending on the experimental setup, single or repeated intravitreal injections were performed.

### 2.4. Tracing and Quantification of Tectal Reinnervation

To evaluate optic nerve regeneration, anterograde biocytin tracing was used as previously described [20, 21, 31]. Briefly, fish were anesthetized in 0.02% buffered tricaine. The left optic nerve was transected between the crush site and the optic nerve head. Next, a piece of gelatin foam presoaked in biocytin (Sigma-Aldrich) was placed on the distal nerve end. After a recovery period of three hours to allow anterograde transport of biocytin along the regenerated RGC axons, fish were euthanized in 0.1% buffered tricaine and transcardially perfused with phosphate-buffered saline (PBS) and 4% paraformaldehyde (PFA). Brains were dissected, fixed overnight in 4% PFA, and embedded in 4% agarose in PBS. Transversal vibratome sections of 50 *μ*m thickness were made. On sections containing the central optic tectum, the biocytin signal was visualized via the Vectastain ABC kit (Vector Laboratories, Burlingame, CA), with diaminobenzidine as chromogen. Next, brain sections were mounted on gelatin-coated glass slides and counterstained for neutral red, allowing brain nucleus visualization. Images were acquired using a microscope Zeiss Imager Z1 at 10x magnification. Quantification of tectal reinnervation was performed using an automated ImageJ script, as previously described [21]. Briefly, the total area innervated by RGC axons (i.e., the stratum fibrosum et griseum superficiale (SFGS) and the stratum opticum (SO) of the optic tectum) was delineated and a threshold was set to measure the biocytin^+^ area within this total area. Axonal density was defined as the ratio between these two values. In naive (uninjured) fish, axonal density was considered maximal and set as a 100% reference. Values of all experimental conditions were expressed relative to this reference. At least six fish were used per condition, and a minimum number of three tectal sections were analyzed per fish.

### 2.5. Visualization and Quantification of the Inflammatory Response

To assess the inflammatory response in the retina, fish were euthanized in 0.1% buffered tricaine at defined time points, depending on the experimental setup. Eyes were removed and fixed for 1 hour in 4% PFA. Then, retinas were dissected and postfixed for 1 hour in 4% PFA. Direct imaging of eGFP^+^/DsRed^+^ cells on retinal whole mounts was performed with an Olympus FV1000 confocal microscope at 20x magnification. To quantify the number of GFP^+^ cells, eight counting frames of 300 *μ*m × 300 *μ*m were used (2 frames in each retinal quadrant, of which 1 central and 1 peripheral). Data were averaged per retina. For retinal cryosections, the eyes were dissected and fixed overnight in 4% PFA. Serial sagittal cryosections (10 *μ*m) were stained with mouse anti-GFP (1 : 250, Millipore, MAB3580), using an Alexa-488 conjugated secondary antibody (1 : 200, Invitrogen). Imaging was performed with a Zeiss Imager Z1 at 40x magnification. To visualize the inflammatory response in the optic tectum, brains were dissected and fixed overnight in 4% PFA. Transversal vibratome sections of the central optic tectum were made (50 *μ*m thickness) and stained with mouse anti-GFP antibody (1 : 250, Millipore, MAB3580), using an Alexa-488 conjugated secondary antibody (1 : 200, Invitrogen). Images were acquired using an Olympus FV1000 confocal microscope at 20x magnification. For quantification, GFP^+^ cells were counted in the SO and SFGS, normalized for the total SO + SFGS area, and averaged per fish.

### 2.6. RNA Isolation and qRT-PCR

Quantitative reverse transcriptase polymerase chain reaction (qRT-PCR) was performed to assess cytokine expression in retinal tissue. Fish were euthanized in 0.1% buffered tricaine, and retinas were dissected and snap frozen. After homogenization in TRI Reagent (Sigma-Aldrich), total RNA was extracted using the NucleoSpin RNA isolation kit (Macherey-Nagel, Germany), according to the manufacturer's instructions. First-strand cDNA was synthetized using oligo dT primers and SuperScript III Reverse Transcriptase (Invitrogen, Belgium). Quantitative PCR reactions were run on a StepOne Plus Real-Time PCR system (Applied Biosystems), using target-specific primers (Table 1) and SYBR Green Master Mix (Applied Biosystems). Per experimental condition, four to seven independent samples were analyzed (consisting of pools of three to four retinas each). All reactions were run in duplicate. geNorm (qBase software [32]) was used to select hypoxanthine phosphoribosyltransferase 1 (hprt1) and succinate dehydrogenase complex subunit A flavoprotein (sdha) as reference genes. Expression levels were calculated using qBase software, which uses an advanced quantification model based on the ΔΔCt method [33].

### 2.7. Statistical Analysis

GraphPad Prism 7 software was used for all statistical analyses. All data were tested for normality (Shapiro-Wilk test) and homoscedasticity (*F* test). Tectal reinnervation data and cytokine expression levels were analyzed using Student's *t*-tests. A probability level (*p*) of <0.05 was considered statistically significant. All data are represented as mean ± SEM. The number of biologically independent samples in each group (*N*) is indicated in the figure captions.

## 3. Results

### 3.1. Systemic Immunosuppression Hampers Optic Nerve Regeneration

**Figure 1:**
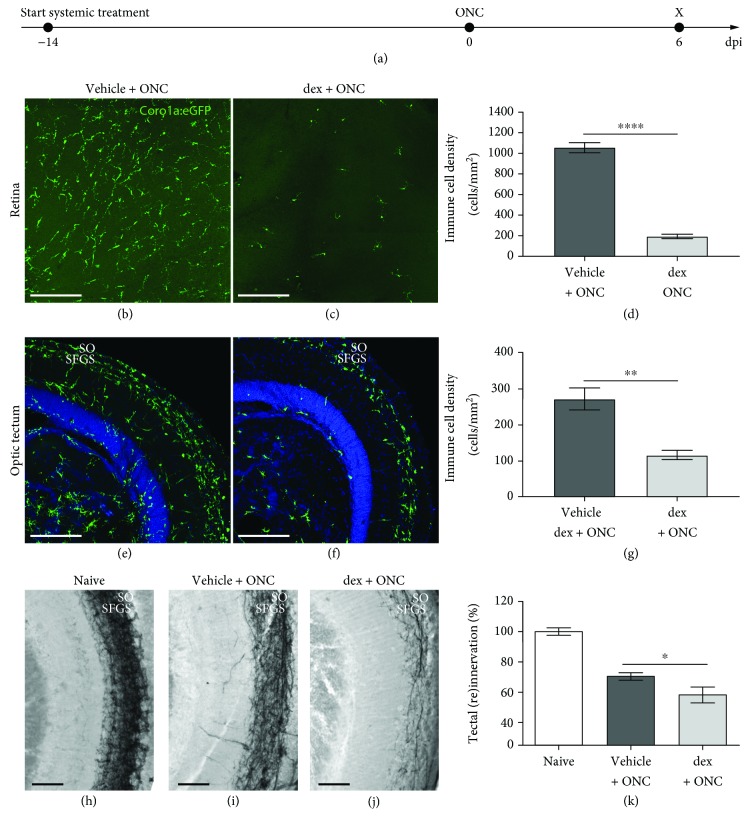
Systemic treatment with dex efficiently depletes microglia/macrophages and restricts optic nerve regeneration. (a) Schematic representation of the experimental setup. The systemic treatment with dex is started two weeks before ONC and is continued until the end of the experiment. The inflammatory response and tectal reinnervation are assessed at 6 dpi. (b-d) Prolonged systemic treatment with dex drastically reduces the number of microglia/macrophages in the retina at 6 dpi (*t*-test, ^∗∗∗∗^*p* ≤ 0.0001). (e-g) At 6 dpi, microglia/macrophages gather in the superficial tectal layers (i.e., the stratum opticum (SO) and the stratum fibrosum et griseum superficiale (SFGS)) in vehicle-treated crushed fish. After systemic dex treatment, a similar organization of microglia/macrophages can be observed, although their number is highly reduced (*t*-test, ^∗∗^*p* ≤ 0.01). (h-j) Representative images of biocytin-traced axons in the optic tectum of naive, uninjured fish (h) and crushed fish treated with vehicle (i) or dex (j), at 6 dpi. (k) Quantification of the regenerating RGC axons revealed that systemic immunosuppression significantly reduces tectal reinnervation at 6 dpi (*t*-test, ^∗^*p* ≤ 0.05). Scale bars: 50 *μ*m. Values represent mean ± SEM. *N* = 6-12.

In a first approach, we studied the effect of systemic immunosuppression on optic nerve regeneration, using the anti-inflammatory drug dex. Hereto, dex was added to the fish tank water and refreshed daily, guaranteeing continuous exposure to the drug. The efficiency of this treatment paradigm has previously been proven in the adult zebrafish CNS [22]. To ensure maximal depletion of microglia/macrophages, the immunosuppressive treatment was started two weeks prior to ONC and continued throughout the following regenerative process. At 6 days post-injury (dpi), a clear decrease in the number of microglia/infiltrating macrophages was observed in the retina, as well as in the optic tectum (Figures 1(a)-1(e)), confirming effective immunosuppression. Quantification of tectal reinnervation revealed a significant decrease in fish treated with dex compared to the vehicle group (Figures 1(f)-1(i)). Thus, systemic immunosuppression negatively affected optic nerve regeneration in zebrafish. Of note, an unusually high mortality rate was observed in the dex-treated fish. Only 40% of the fish in this group (6 out of 15 fish) were still alive at the end of the experiment, compared to 92% (12 out of 13 fish) in the vehicle-treated group. Conclusively, we demonstrated that systemic immunosuppression with dex efficiently depletes microglia/macrophages in the retina and the optic tectum and hampers optic nerve regeneration, although the latter may be partly attributed to a general non-well-being of the fish.

### 3.2. Intravitreal Dex Administration Induces Local and Temporary Immunosuppression

**Figure 2:**
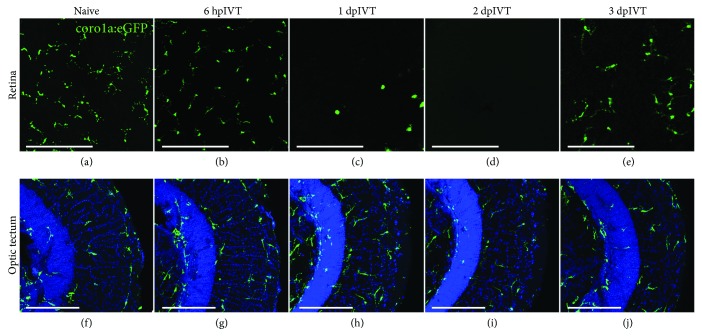
Local treatment with dex efficiently depletes microglia/macrophages in the inner retina and leaves the optic tectum unaffected. (a-e) A single intravitreal injection of dex causes a transient depletion of retinal microglia in the inner retina. At 6 hpIVT (b), microglia are still present, but they seem to have retracted their processes. At 1 and 2 dpIVT (c-d), the inner retina is almost devoid of microglia, while at 3 dpIVT (e), repopulation of the retina is ongoing. (f-j) Intravitreal injection of dex does not affect the microglial number or their appearance in the optic tectum. The outer tectal layers, i.e., the stratum opticum (SO) and the stratum fibrosum et griseum superficiale (SFGS), are indicated. Scale bars: *50 μ*m.

To avoid the confounding effects of systemic immunosuppression with dex, we turned to an alternative experimental setup, where dex was delivered locally via intravitreal injection. First, we studied the efficiency of this local application in the uninjured retina. At 6 hours post-intravitreal injection (hpIVT) of dex, the number of retinal microglia was not yet decreased, but microglia seemed to have retracted their processes. We observed depletion of microglia in the inner retina from 1 day post-injection (dpIVT) onwards, lasting until day 2. In line with previous reports [34], the microglial depletion was transient, as at 3 days after dex injection, microglia and/or infiltrating macrophages started to repopulate the retina (Figures 2(a)-2(e)). Of note, microglia in the optic tectum (or adjacent brain regions) were not affected by the intravitreal dex treatment, confirming its local effect (Figures 2(f)-2(j)). We thus conclude that short-term depletion of retinal microglia can be efficiently achieved via intravitreal dex administration.

### 3.3. Local Immunosuppressive Treatment Induces an Exaggerated Inflammatory Response to Subsequent Optic Nerve Injury

**Figure 3:**
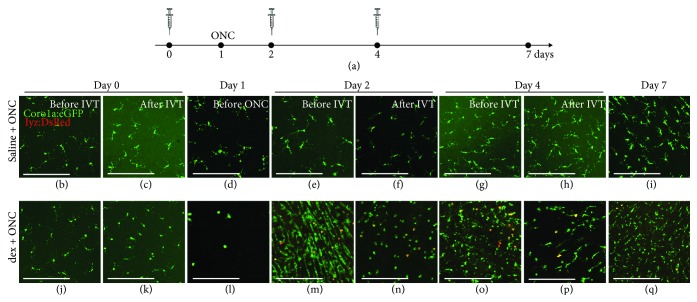
Local immunosuppressive treatment with dex induces an exaggerated inflammatory response to subsequent optic nerve injury. (a) Schematic representation of the experimental setup. Intravitreal injection (IVT) of dex (or saline) is performed three times, at days 0, 2, and 4. ONC is performed at day 1. Retinas are harvested just before and at 6 hours after each injection (days 0, 2, and 4), just before the moment of ONC (day 1), and at day 7, to assess their number and morphology in the inner retina. (b-i) Intravitreal injection of saline has no effect on the retinal microglia appearance before or after ONC. However, from day 4 onwards (i.e., 3 dpi), a restricted increase in the number of microglia/macrophages can be observed, as we have shown before [21]. (j-q) As a result of the first intravitreal dex injection at day 0, the inner retina is almost completely devoid of microglia at the moment of ONC (day 1). Nevertheless, at day 2 (before the second injection), the number of microglia/macrophages increases drastically, compared to the previous time points, and to the saline + ONC fish. Neutrophil infiltration is observed as well (yellow). This increase is partly counteracted by the second dex injection. Indeed, after the injection at day 2, the number of microglia/macrophages and neutrophils is reduced. At day 4, the number of innate immune cells increased again, and subsequent dex injection temporarily decreased this response once more. At day 7, the number of microglia/macrophages is again higher than right after the injection at day 4 in the dex + ONC group. Additionally, there are still more innate immune cells in the dex + ONC than in the saline + ONC group at 7 days post-ONC. Scale bars: *50 μ*m.

In order to study the effect of local immunosuppression on optic nerve regeneration, the depletion should be prolonged and maintained throughout the regenerative process. Therefore, multiple injections were performed: dex (or saline) was applied intravitreally for three times in total, every two days (i.e., at days 0, 2, and 4). Fish underwent ONC at day 1, and the inflammatory response in the retina was assessed at multiple time points during the experiment (Figure 3). As expected, no effect of intravitreal saline injections on the retinal microglia/macrophage cell number or on their morphology was observed. Notably, however, an increase in inflammatory cells could be observed after ONC, confirming previous studies where a transient elevation of the number of microglia/macrophages was found, peaking around 7 dpi [21, 24]. In contrast, an apparent effect on retinal microglia/macrophages was found in the dex-treated (“dex + ONC”) group. Upon the first intravitreal injection, microglial depletion was obtained after 1 day, validating the results described above. Thus, at the moment of ONC, the retina was almost completely devoid of microglia. Surprisingly however, at day 2 (i.e., at 1 day post-ONC), we noticed a massive increase in the number of microglia/macrophages in the inner retina, as well as a prominent infiltration of neutrophils. Subsequent intravitreal injection of dex at the same day decreased the inflammatory cell number again, confirming the depletion capacity of dex. Nevertheless, there were still more innate immune cells in the retina of dex + ONC fish, as compared to the uninjured retina and compared to saline + ONC fish at the same time point after optic nerve injury. In addition, microglia/macrophages showed an amoeboid morphology, indicative of their reactivation. At day 4, the number of microglia/macrophages and neutrophils was found to be elevated again. This response could again be lessened as a small decrease in innate immune cells was observed at 6 h after dex application at day 4. Of note, however, treatment with dex resulted in a higher number of innate immune cells than in the saline + ONC group at day 7 (i.e., 6 dpi). Overall, our data revealed that multiple dex injections in the regenerating retina do not result in sustained immunosuppression. Instead, we observed an enhanced inflammatory response to ONC in dex-treated fish, which cannot be fully suppressed by later dex application.

**Figure 4:**
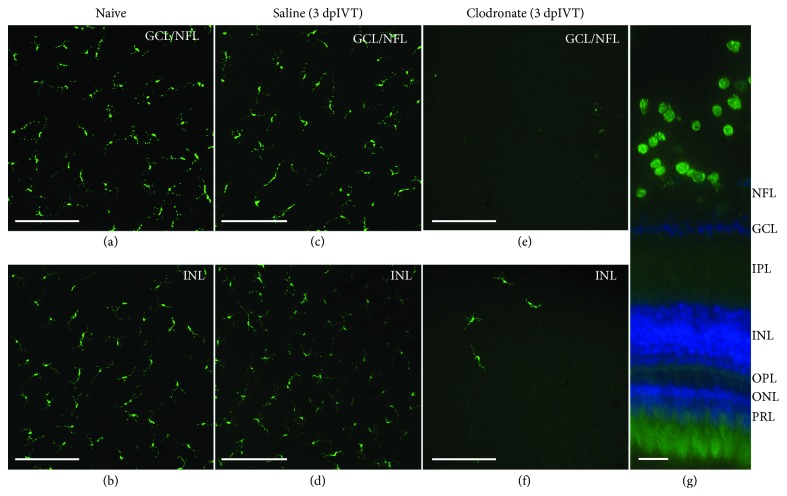
Local treatment with clodronate liposomes efficiently depletes microglia/macrophages in the retina. Retinal microglia/macrophages are assessed in naive fish and after a single intravitreal injection of either saline or clodronate liposomes (3 dpIVT). Different focal planes are used to show microglia on retinal flat mounts in the innermost retina (GCL/NFL) and in the INL, respectively. (a-d) A single intravitreal injection of saline does not affect the retinal microglial morphology or number, as compared to naive uninjected fish. (e-g) At 3 days after intravitreal injection of clodronate liposomes, retinal microglia migrate towards the vitreous, leaving the retina almost completely depleted, as can be seen on retinal flat mounts (e-f), and is even more obvious in a retinal cross-section (g). Scale bars: *50 μ*m (a-f); 20 *μ*m (g). GCL: ganglion cell layer; INL: inner nuclear layer; IPL: inner plexiform layer; NFL: nerve fiber layer; ONL: outer nuclear layer; OPL: outer plexiform layer; PRL: photoreceptor layer.

**Figure 5:**
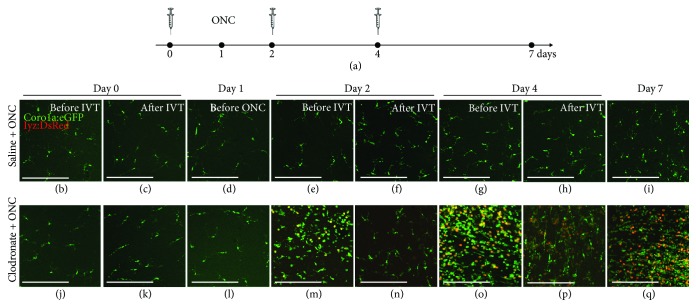
Local immunosuppressive treatment with clodronate liposomes induces an exaggerated inflammatory response to subsequent optic nerve injury. (a) Schematic representation of the experimental setup. Intravitreal injection of clodronate liposomes (or saline) is performed three times, at days 0, 2, and 4, and ONC is performed at day 1. The inflammatory response is assessed just before and 6 h after each injection (days 0, 2, and 4), just before the moment of ONC (day 1), and at day 7. (b-i) Intravitreal injection of saline does not affect retinal microglial appearance before nor after ONC. A restricted increase in the number of microglia/macrophages can be observed from day 4 onwards (i.e., 3 dpi), as we have shown before [21]. (j-q) Although full microglial depletion is not yet obtained at day 1, ONC induces a prominent increase in the number of retinal microglia/macrophages at day 2 (before the second injection) in the clodronate + ONC retinas, as compared to saline + ONC retinas. Neutrophil infiltration is apparent as well in the clodronate + ONC group at this point. This inflammatory reaction is partially counteracted by subsequent application of clodronate liposomes at day 2. However, this suppression is clearly temporary as the number of innate immune cells is again massively increased before the third clodronate injection at day 4. Again, this striking inflammatory response can be blocked by a third clodronate injection. Still, at day 7 the number of microglia/macrophages is much higher than seen in the saline + ONC group at the same time point. Scale bars: *50 μ*m.

Since these observations were unexpected, we used clodronate liposomes as an alternative pharmacological method for microglial/macrophage depletion [35, 36] in order to validate our results. First, we confirmed the depletion capacity of clodronate liposomes in the uninjured zebrafish retina. Mirroring the results described above, intravitreal injection of clodronate liposomes efficiently decreased the number of microglia in the uninjured retina (Figure 4). The retina was almost devoid of microglia at 3 dpIVT, highly comparable to the results we obtained in the dex model described above. Subsequently, multiple intravitreal injections of clodronate liposomes were combined with optic nerve injury. We used the same experimental setup as for dex, encompassing three intravitreal injections (at days 0, 2, and 4) and performed ONC at one day after the first injection. With this treatment paradigm, we obtained similar results as described above for dex: repetitive intravitreal clodronate injections resulted in alternating waves of microglia depletion and exaggerated inflammatory responses. Mirroring the results described above, this inflammatory reaction surpassed by far the response in saline + ONC fish (Figure 5). We can thus conclude that in both models, the local immunosuppressive treatment induces an exaggerated inflammatory response in the retina after optic nerve injury, which cannot be completely abrogated using repeated administration of the immunosuppressors.

### 3.4. Local Immunosuppressive Treatment Induces Accelerated Tectal Reinnervation after Optic Nerve Injury

**Figure 6:**
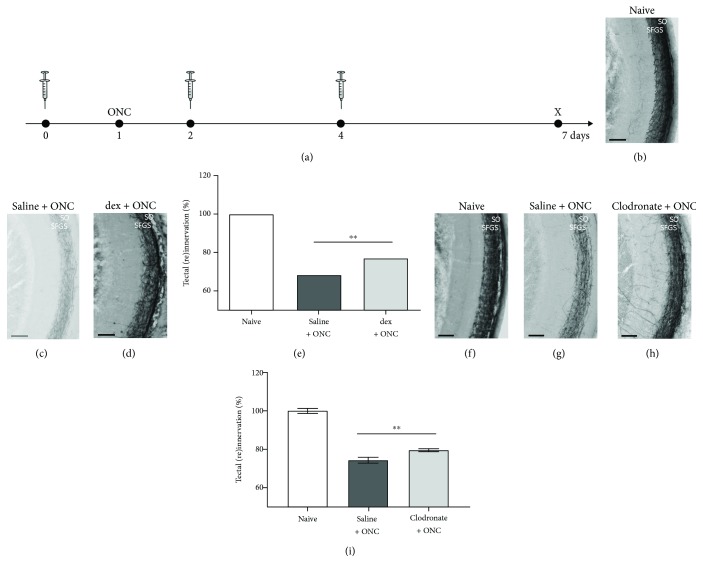
Multiple intravitreal injections of dex or clodronate liposomes accelerate tectal reinnervation after optic nerve injury. (a) Schematic representation of the experimental setup. Intravitreal injection of dex or clodronate liposomes (or saline) is performed three times, at days 0, 2, and 4, and ONC is performed at day 1. Tectal reinnervation is assessed at day 7 (i.e., 6 dpi). (b-e) Repeated intravitreal injections of dex induce significantly higher tectal reinnervation in the dex + ONC group as compared to the saline + ONC group (*t*-test, ^∗∗^*p* ≤ 0.01). (f-i) Similarly, representative pictures and quantification of tectal reinnervation show a significant increase after treatment with clodronate liposomes compared to saline + ONC fish (*t*-test, ^∗∗^*p* ≤ 0.01). Scale bars: *50 μ*m. Values represent mean ± SEM. *N* = 11-14 (b-e), *N* = 9 (f-i).

Next, we studied how the augmented inflammatory response, engendered by multiple intravitreal dex injections, affected the regenerative process. Hereto, dex was administered, following the same experimental setup as described above, i.e., injections at days 0, 2, and 4. Fish underwent optic nerve injury at one day after the first injection. Tectal reinnervation was assessed via anterograde biocytin tracing one week after the first injection (i.e., at 6 dpi) (Figure 6(a)). We found a significantly higher number of regenerated axons in the optic tectum of dex + ONC fish treated with dex, compared to saline + ONC fish (76.99 ± 1.64% versus 68.39 ± 2.30%, respectively) (Figures 6(b)-6(e)). This enhanced regenerative response was confirmed using clodronate liposomes: quantification of tectal reinnervation at 6 dpi revealed an increase in the innervated area, as compared to the saline + ONC group (79.58 ± 0.80% versus 74.38 ± 1.45%, respectively) (Figures 6(f)-6(i)). As such, it is clear that the increased inflammatory response is accompanied by accelerated optic nerve regeneration. This accelerated regeneration contrasts our results after systemic immunosuppression, but is in line with the premise that an increased inflammatory response can stimulate optic nerve regeneration.

### 3.5. The First Exposure to Dex Induces Immune Cell Sensitization in the Retina

**Figure 7:**
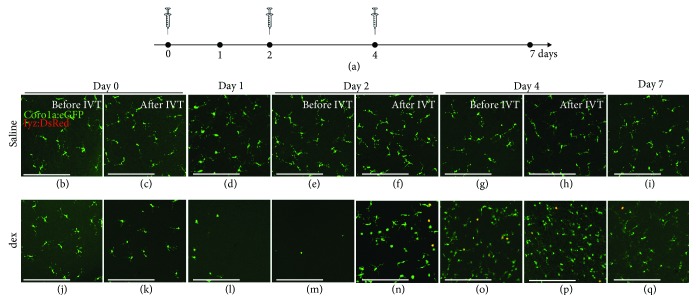
Multiple intravitreal injections with dex cause an inflammatory response in the retina. (a) Schematic representation of the experimental setup. Intravitreal injection (IVT) of dex (or saline) is performed three times, at days 0, 2, and 4. Retinas are harvested just before and at 6 hours after each injection (days 0, 2, and 4), at days 1 and 7, to assess their number and morphology in the inner retina. (b-i) Intravitreal injection of saline does not alter the number of retinal microglia, or their morphology, at any of the time points under study. (j-q) As a result of the first dex injection (at day 0), the retina is almost completely devoid of microglia at days 1 and 2. Yet, surprisingly, the second dex injection (at day 2) induces an increase in the number of retinal microglia/macrophages and the infiltration of neutrophils (yellow). Subsequent application of dex (at day 4) has a similar effect: it increases rather than decreases the number of microglia/macrophages in the retina. At day 7, the inflammatory status seems to have normalized again. Scale bars: *50 μ*m.

The enhanced inflammatory response upon ONC observed after local immunosuppressive treatment might be explained by microglial/macrophage sensitization, which subsequently results in an exaggerated reaction to optic nerve injury. To investigate this hypothesis, we first studied how the uninjured retina responds to multiple dex injections without optic nerve injury. Hereto, dex administration was performed following the same scheme as described above, i.e., three consecutive intravitreal injections at days 0, 2, and 4, without performing ONC at day 1. The number and morphology of retinal microglia was assessed at multiple time points during the experiment, namely, at days 0, 1, 2, 4, and 7 (Figure 7). As expected, intravitreal application of saline did not affect the number of the retinal microglia, or their morphology. The first dex injection, in contrast, induced depletion of retinal microglia, again confirming the results described above. Yet, microglial depletion was not maintained after the following dex injections, and instead an increase in the number of microglia/macrophages could be observed soon after the second injection. Most of them displayed an amoeboid morphology. In addition, some neutrophils had infiltrated the retina as well. It is thus clear that also in the uninjured retina, the depletion effect of a single dex injection cannot be prolonged by subsequent intravitreal administration 2 and 4 days later. Instead, the first exposure to dex seemed to induce microglial sensitization (at least in those cells that survive the treatment) and as a consequence, the subsequent intravitreal injection, which can be considered a secondary immune challenge, induced an inflammatory response in the retina. Both the intravitreal dex injection and optic nerve injury can thus be regarded as a secondary immune challenge; however, comparison of Figures 3(m)–7(n) shows that the immune response evoked by dex injection was smaller than that induced by dex injection combined with optic nerve injury.

**Figure 8:**
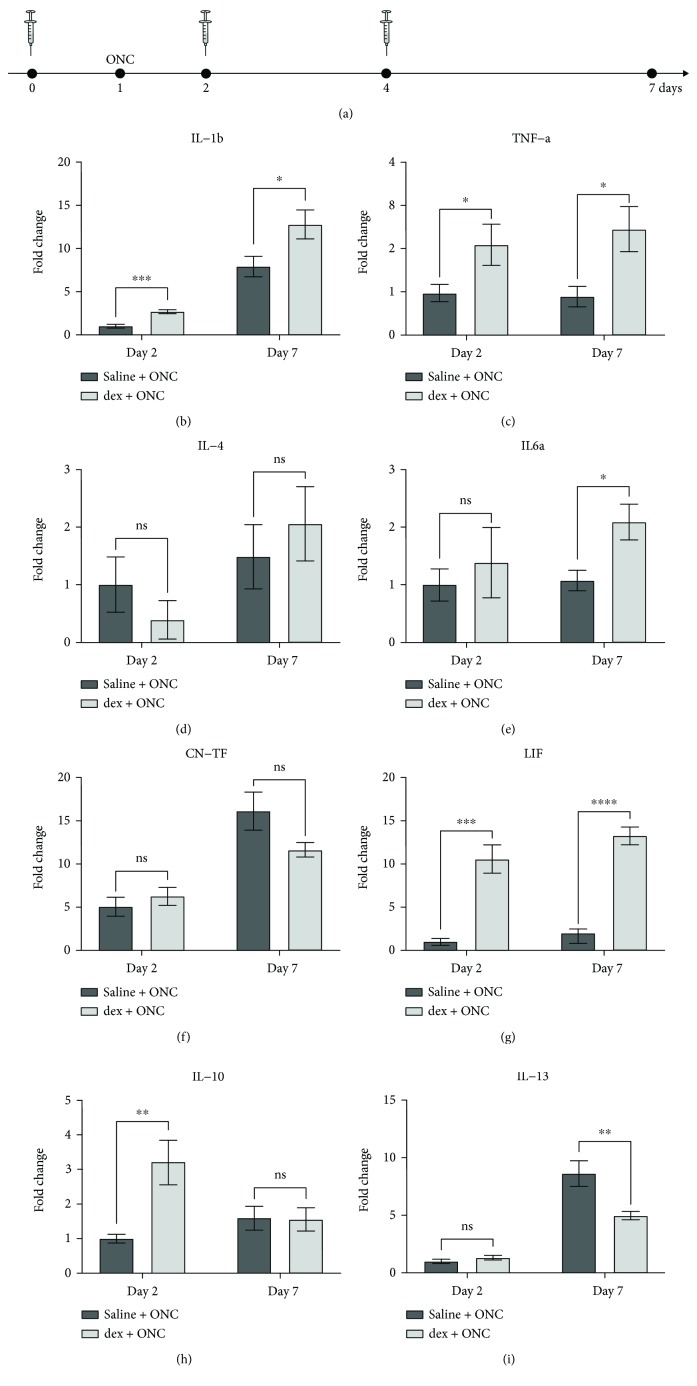
Local treatment with dex increases the retinal cytokine expression. (a) Schematic representation of the experimental setup. Intravitreal injection (IVT) of dex (or saline) is performed three times, at days 0, 2, and 4. ONC is performed at day 1. Retinal cytokine expression is assessed via qRT-PCR at day 2 (before the injection) and at day 7. (b-i) Quantification reveals a significant increase in the expression of IL-1*β*, TNF-*α*, and LIF at both days 2 and day 7 upon dex + ONC treatment compared to the saline + ONC group. For IL-6 and IL-10, the increase is significant only at day 7 or day 2, respectively. IL-13 shows a significantly reduced expression at day 7 in dex + ONC fish. The expression levels of IL-4 and CNTF remain unchanged (*t*-tests, ^∗^*p* ≤ 0.05, ^∗∗^*p* ≤ 0.01, ^∗∗∗^*p* ≤ 0.001, and ^∗∗∗∗^*p* ≤ 0.0001). Values represent mean ± SEM. *N* = 4-7.

To further confirm retinal microglia/macrophage sensitization in dex-treated fish, we investigated whether the cytokine expression in the retina differs in ONC + saline versus combined ONC + dex fish, as augmented cytokine levels are considered a molecular signature of the exaggerated inflammatory response upon sensitization [37–40]. Hereto, dex (or saline) was again administered at days 0, 2, and 4, and ONC was performed at day 1. Retinal samples were harvested at day 2 (i.e., 1 dpi, when the sensitization effect was most apparent) and day 7 (i.e., 6 dpi, the time point where tectal reinnervation was assessed). The expression of a selected set of cytokines was determined via qRT-PCR. First, we found that dex + ONC treatment causes a significant increase in expression of the proinflammatory cytokines interleukin- (IL-) 1*β*, tumor necrosis factor *α* (TNF-*α*), and leukemia inhibitory factor (LIF) at day 2 (fold changes of 2.7, 2.1, and 10.6, respectively) and day 7 (fold changes of 1.6, 2.7, and 6.5, respectively) in comparison with the saline + ONC condition. Second, the increase in IL-10 expression was found to be transient, with a significant 3.2-fold increase at day 2, but with expression levels close to the ONC + saline group at day 7. IL-6 expression showed the opposite: while its expression level was still similar to the ONC + saline group at day 2, it had increased at day 7 in dex-treated animals. Lastly, mRNA levels of IL-4 and CNTF remained unchanged at day 2 as well as at day 7 (Figure 8). Thus, although not all are affected in the same way, it is clear that repeated dex administration has an effect on cytokine expression in the damaged retina. Together with the morphological findings described above, these data indicate that local immunosuppression causes microglial/macrophage sensitization, which results in an exaggerated (pro)inflammatory response to secondary immune challenges, such as optic nerve injury.

## 4. Discussion

In this study, we addressed the feasibility of pharmacological immunosuppression in the zebrafish visual system and investigated its effects on optic nerve regeneration. First, we used a systemic immunosuppressive treatment paradigm that has previously been shown to efficiently suppress the immune system in adult zebrafish [22]. We verified that in the ONC model the number of inflammatory cells was greatly reduced in the retina as well as in the optic tectum and revealed that systemic immunosuppression restricts optic nerve regeneration. This is in line with previous studies in other zebrafish regeneration models. Indeed, after traumatic brain injury, systemic treatment with dex was reported to significantly reduce the reactive proliferation of radial glial cells and subsequent neurogenesis, both of which are indispensable for successful neuronal regeneration [22]. Furthermore, systemic administration of glucocorticoids was shown to hamper tail fin regeneration [22, 41] and cardiac repair [42]. Overall, these results suggest that the inflammatory response upon acute injuries beneficially contributes to successful regeneration in various zebrafish models, including optic nerve injury. However, we observed an unusually high mortality rate in dex-treated fish after optic nerve injury, which has not been reported previously. The reason for this is unclear, but may be attributed to the long treatment time in our model (2 weeks of pretreatment and 6 days of regeneration) and/or differences in housing and feeding conditions. Anyhow, part of the observed outcome after systemic dex treatment may thus arise from the general non-well-being of the fish and highlight that care should be taken when interpreting regenerative results from systemic immunosuppression studies.

In addition to systemic immunosuppression, we studied the effects of local dex and clodronate liposome administration in the visual system, which can be performed easily via intravitreal injection. We showed that a single injection efficiently depletes retinal microglia in the uninjured retina. Intravitreal administration of clodronate liposomes was found to induce migration of retinal microglia towards the liposome bodies in the vitreous. Upon phagocytosis of the liposomes, they are exposed to the toxin clodronate, which eventually causes apoptotic cell death [35, 43]. Although we did not investigate the precise underlying mechanism of the microglial depletion in the dex model, we hypothesize that it also occurs via apoptosis [44–47]. Importantly, however, the depletion is transient. This finding is consistent with previous findings in rodent models, where rapid repopulation of the retina is observed upon cessation of immunosuppressive treatment [48–55]. Studies in rodents disclosed that under normal conditions, infiltration of peripheral bone marrow-derived cells is highly restricted and thus cannot account for the replenishment of the microglial pool [49, 56, 57]. Instead, microglial repopulation most likely occurs through local proliferation. Elmore and coworkers have suggested the existence of previously undescribed microglial progenitor cells in the CNS, which may account for replenishment of the microglial pool [48]. Others have argued against this rather controversial view and propose that newly generated microglia arise from hyperproliferation of the few surviving cells [51, 58]. This is supported by the notion that microglia can show high turnover rates already in steady-state conditions [59]. Alternatively, the new microglial population may be generated from nearby extraretinal sources, such as the cells residing in the optic nerve [58]. In zebrafish, the capacity of the microglial cell population to renew itself has not yet been studied in detail. Although our results parallel the rapid replenishment of the microglial pool in rodents, the origin of the repopulating microglia in our zebrafish depletion models awaits to be investigated. Also, further studies are needed to determine whether the newly generated microglia are morphologically and functionally indistinct from the original microglial cells. Importantly, as a consequence of the transient nature of local microglial depletion, repeated drug administration is necessary to enable the study of the effect of continued local immunosuppression. Unfortunately, we were unable to achieve sustained immunosuppression through multiple intravitreal injections of dex or clodronate liposomes, neither in the uninjured nor in the regenerating retina. A possible explanation for this is the relatively rapid washout of the immunosuppressive drugs from the vitreous, wherefore they are not continuously present during the experiment. Most likely, sustained immunosuppression during optic nerve regeneration would require more frequent drug administration, for which methods other than intravitreal injection are needed. Indeed, performing more intravitreal injections increases the risk to injure the eye and the retina, thereby creating confounding effects, such as an inflammatory response upon retinal damage.

In contrast to the expected depletion of microglia/macrophages upon multiple intravitreal injections of immunosuppressive drugs, our local immunosuppression treatment paradigm stimulated the inflammatory response upon intravitreal injection. In the uninjured retina, a rapid increase in the number of microglia/macrophages in the retina, as well as neutrophil infiltration, is observed upon the second dex or clodronate injection. Likewise, an inflammatory reaction is observed soon after optic nerve injury in dex- or clodronate-treated fish, which outweighs the normal inflammatory response to ONC. This result may be surprising at first sight, since both compounds are generally considered as immunosuppressive. We hypothesize, however, that the incomplete microglial depletion upon dex or clodronate exposure can be considered as a trigger provoking the retina, after which surviving microglia and/or recruited blood-borne macrophages develop a sensitized (or primed) profile. This explains the exaggerated inflammatory response to optic nerve injury after prior treatment with dex or clodronate liposomes. In support of this sensitization hypothesis, and similar to previous studies in rodents on immune cell sensitization [38–40, 60], we showed augmented expression of the proinflammatory cytokines IL-1*β*, IL-6, and TNF-*α* in dex-treated zebrafish retinas. Additionally, we found an early significant increase in the expression of the anti-inflammatory cytokine IL-10, which may possibly act as a balancer to counteract the excessive retinal inflammation [61]. Therefore, we hypothesize that the first intravitreal dex injection resulted in incomplete immunosuppression and induced sensitization of the microglia/macrophages. Importantly, studies in rodents have provided evidence for immune cell sensitization upon treatment with glucocorticoids (such as dex) under certain circumstances. The relative timing of glucocorticoid exposure and the immune challenge seems to be one of the major factors determining the inflammatory outcome. Indeed, while (systemic) glucocorticoid administration demonstrates its well-known anti-inflammatory effects when applied *after* an immune challenge (such as treatment with lipopolysaccharide (LPS)), *prior* glucocorticoid exposure was found to induce immune cell sensitization. Consequently, the inflammatory response to subsequent LPS treatment was potentiated, demonstrated by an even stronger increase in TNF-*α*, IL-1*β*, and IL-6 mRNA levels [40]. Most likely, in the current study, the first intravitreal dex injection sensitized the retinal microglia, at least those that survived the suppressive treatment. As a consequence, they show an exaggerated response to ONC (or to the second dex injection in the uninjured retina).

Interestingly, we also demonstrated that the retinal expression of LIF was upregulated upon immune cell sensitization. LIF has been suggested to be involved in the regenerative process after optic nerve injury in zebrafish [25]. As such, its augmented expression may be linked to the accelerated tectal reinnervation that we observed after repeated local dex administration. This is in line with previous reports showing that increasing the inflammatory status improves the regenerative outcome after optic nerve injury, in mammals as well as in zebrafish [24, 62–64]. Indeed, neuroinflammation has been proposed as a pivotal player in CNS regeneration [22, 63–66]. In sum, this study adds to the existing evidence for a regeneration-promoting role of acute inflammation.

To confirm our findings, we employed clodronate liposomes as an alternative immunosuppressive method. Parallel to local treatment with dex, we found that repeated administration of clodronate liposomes could not engender sustained depletion of microglia/macrophages in the retina, despite effective immunosuppression upon single exposure in the uninjured retina. Instead, we observed an exaggerated response to ONC in clodronate-treated fish as well as enhanced optic nerve regeneration, mirroring our findings in the dex-injected animals. These results indicate that the first exposure to clodronate liposomes may also induce immune cell sensitization, similar to what we demonstrated in the dex model. Interestingly, local intravitreal administration of clodronate liposomes has also been used previously in rodent models to study the effect of macrophage (and microglial) depletion on the regenerative outcome after optic nerve injury [67, 68]. Here, significant depletion of retinal microglia/macrophages was demonstrated at 1-3 weeks later. This local immunosuppressive treatment thus seems effective in the regenerating rodent retina, contrasting our results in zebrafish. Notably, in these studies, the first administration of clodronate liposomes was performed at the same day of optic nerve damage and microglia/macrophages were thus not yet depleted at the moment of injury [67, 68]. Interestingly, the local depletion of microglia/macrophages did not impede RGC regeneration [67], and one study reports that the treatment with clodronate liposomes even increases the number of regrowing axons [68]. Whether sensitization of microglia/macrophages at early time points after optic nerve injury contributes to this effect remains unclear. Additionally, concurrent systemic depletion of microglia and macrophages, respectively, via colony-stimulating factor 1 receptor (CSF1R) inhibition and clodronate liposomes was found to compromise optic nerve regeneration in rodents only to a limited extent, suggesting that microglia and macrophages are not critically involved in the regenerative process [9]. These results contrast our current findings in zebrafish, as we show that acute inflammatory processes do seem to play a role in optic nerve regeneration. As such, it is clear that further study on the cellular and molecular (inflammatory) mechanisms underlying optic nerve regeneration is required.

Notably, these findings are reminiscent of the well-described concept of chronic microglial sensitization or “priming.” As a result of variable CNS insults (such as traumatic brain injury, neurodegenerative disease, or aging), microglia may develop a primed profile, which is characterized by a higher baseline expression of inflammatory markers and a lower threshold to become reactivated. Consequently, the microglial response to a secondary insult is exaggerated and prolonged compared to their nonprimed counterparts. Depending on the context, microglial priming can lead to CNS dysfunction and neurobehavioral complications [37, 69, 70]. Our observation of microglial sensitization after local immunosuppression may be considered analogous to this concept of priming, as we demonstrate that immune cell sensitization affects the regenerative outcome after ONC. Yet, in our zebrafish model, the effect is positive and leads to enhanced regeneration. Thus, further study on the mechanisms that underlie this preconditioning, and how the inflammatory machinery can be instructed to provide the appropriate context for CNS regeneration, is highly warranted.

## 5. Conclusion

In this study, we disclosed a strong positive correlation between the acute inflammatory response in the retina and the regenerative capacity of the optic nerve in zebrafish subjected to nerve injury. We first showed that systemic immunosuppression with dex hampers the regenerative process. Secondly, we demonstrated that local application of dex or clodronate liposomes prior to injury can induce immune cell sensitization in the retina, which results in an exaggerated inflammatory reaction to optic nerve injury and eventually in enhanced regeneration. Overall, the results from both systemic and local immunosuppression treatments point towards a beneficial role of acute inflammation in zebrafish optic nerve injury.

## Figures and Tables

**Table 1:** Sequences of the primers used for qRT-PCR reactions.

Gene	Primer sequences	Reference
IL-1*β*	Forward 5′-GATCCAAACGGATACGACC-3′	
	Reverse 5′-TGATAAACCAACCGGGACA -3′	
IL-4	Forward 5′-GATCCTGAATGGGAAAGGGG-3′	
	Reverse 5′-GTAGATGAGACCTGCTTGGA-3′	
IL-6	Forward 5′-GCTACACTGGCTACACTCTT-3′	
	Reverse 5′-TCGCCAAGGAGACTCTTTAC-3′	
IL-10	Forward 5′-AGGAACTCAAGCGGGATATG-3′	
	Reverse 5′-GAGGCTAGATACTGCTCGAT-3′	
IL-13	Forward 5′-TTTTACGTTGAAAGGCACGG-3′	
	Reverse 5′-CCTTTGTCTCTTTTGGGGGA-3′	
CNTF	Forward 5′-GCGACTGGTGGGAGTTTTG-3′	[25]
	Reverse 5′-AGCACCTCTTCTTGTCCGTTG-3′	
LIF	Forward 5′-CAAGTCAAATTCAGAGCATACTTCG-3′	[25]
	Reverse 5′-TGAGCTTCAGACTTCGGTGAA-3′	
TNF-*α*	Forward 5′-AGGGCAATCAACAAGATGGA-3′	
	Reverse 5′-GACACCTGGCTGTAGACAAA-3′	
hprt1	Forward 5′-TGGACCGAACTGAACGTCTG-3′	[21]
	Reverse 5′-TGGGAATGGAGCGATCACTG-3′	
sdha	Forward 5′-ACGCACCCAATGCCAAAGAC-3′	[21]
	Reverse 5′-TCTTTATCCGGCCCAACACC-3′	

## Data Availability

The data sets generated and analyzed during the current study are available from the corresponding author on reasonable request.

## Abbreviations

CNS: Central nervous system
CNTF: Ciliary neurotrophic factor
Coro1a: Coronin1a
CSF1R: Colony stimulating factor 1 receptor
DAPI: 4′,6-Diamidino-2-phenylindole
dex: Dexamethasone
dpi: Days post-injury
dpIVT: Days post-intravitreal injection
DsRed: *Discosoma* sp. red fluorescent protein
eGFP: Enhanced green fluorescent protein
FITC: Fluorescein-isothiocyanate
GCL: Ganglion cell layer
GFP: Green fluorescent protein
hpIVT: Hours post-intravitreal injection
hprt1: Hypoxanthine phosphoribosyltransferase 1
IL: Interleukin
INL: Inner nuclear layer
IPL: Inner plexiform layer
IVT: Intravitreal injection
LIF: Leukemia inhibitory factor
LPS: Lipopolysaccharide
NFL: Nerve fiber layer
ONC: Optic nerve crush
ONL: Outer nuclear layer
OPL: Outer plexiform layer
PBS: Phosphate-buffered saline
PFA: Paraformaldehyde
PRL: Photoreceptor layer
qRT-PCR: Quantitative reverse-transcriptase polymerase chain reaction
RGC: Retinal ganglion cell
sdha: Succinate dehydrogenase complex subunit A flavoprotein
SFGS: Stratum fibrosum et griseum superficiale
Sfpq: Splicing factor proline/glutamine-rich
SO: Stratum opticum
TNF-*α*: Tumor necrosis factor alpha
TLR2: Toll-like receptor 2.
